# Intersecting barriers to pursuing clinical academia

**DOI:** 10.1177/01410768211046180

**Published:** 2021-09-20

**Authors:** Temitope Fisayo

**Affiliations:** King's College London, College London Faculty of Life Sciences and Medicine, London SE1 1UL, UK

I commend Curtis-Lopez et al. for their recent commentary piece highlighting the lack of racial diversity in medical academia and the barriers minority-ethnic medical students face.^1^

However, strikingly absent from their piece is the role of class in determining who can succeed in academic medicine. People from an ethnic minority in the UK are disproportionately working class,^2^ so it is likely they will face an additional class barrier to becoming involved in academic medicine.

Evidence is clear that students who attend medical schools with mandatory intercalation are far more likely to apply to the Academic Foundation Programme.^3^ This is the main entry point for medical students interested in a career as a clinical academic. It is cheaper to attend a school with a five-year programme and therefore plausible that more students from a lower socioeconomic background attend these schools.

Many medical students, particularly those from a working-class background, have a part-time job to support themselves. Often, you need to be able to afford to do unpaid research to get a foot in the door of academic research. Time ‘volunteered’ that is spent trawling datasets or conducting qualitative interviews is time away from valuable employment. A PubMed-indexed publication might look impressive during interviews for posts in the future but cannot cover today’s rent.

While some medical schools offer paid opportunities to get involved in research, these are limited and competitive. This means for unsuccessful applicants – who depend on the funds to be able to pursue their research interests – there is little other option. Their wealthier, whiter counterparts do not need to compete to take up kind offers from professors encountered on placement.

How might we tackle the material barriers that prevent medical academia from being more diverse? Routinely recognising the research work medical students routinely perform as *work* – that is, labour entitled to a corresponding wage – might be a good start.

## Declarations

**Ethics approval:** Not applicable.

**Guarantor:** TF.

**Contributorship:** Sole author.

**Acknowledgements:** None.

**Provenance:** Not commissioned; editorial review.

**ORCID iD:** Temitope Fisayo https://orcid.org/0000-0002-3576-3214
